# Lingonberry (*Vaccinium vitis-idaea* L.) Fruit as a Source of Bioactive Compounds with Health-Promoting Effects—A Review

**DOI:** 10.3390/ijms22105126

**Published:** 2021-05-12

**Authors:** Katarzyna Kowalska

**Affiliations:** Department of Biotechnology and Food Microbiology, Poznan University of Life Sciences, 48 Wojska Polskiego St., 60-627 Poznan, Poland; katarzyna.kowalska@up.poznan.pl

**Keywords:** lingonberry fruit, health benefits, anti-inflammatory, antioxidant activity, obesity, diabetes, neurodegenerative disorders

## Abstract

Berries, especially members of the Ericaceae family, are among the best dietary sources of bioactive compounds with beneficial health effects. The most popular berries are in the genus *Vaccinium*, such as bilberry (*Vaccinium myrtillus*), cranberry (*Vaccinium macrocarpon*, *V. oxycoccos*), and blueberry (*Vaccinium corymbosum*). Lingonberry (*Vaccinium vitis-idaea*) is less prevalent in the daily human diet because they are collected from the wild, and plant breeding of lingonberry is still on a small scale. Lingonberries are classed as “superfruits” with the highest content of antioxidants among berries and a broad range of health-promoting effects. Many studies showed various beneficial effects of lingonberries, such as anti-inflammatory, antioxidant, and anticancer activities. Lingonberries have been shown to prevent low-grade inflammation and diet-induced obesity in diabetic animals. Moreover, lingonberry intake has been associated with a beneficial effect on preventing and treating brain aging and neurodegenerative disorders. The consumption of berries and their health-promoting activity is a subject receiving a great deal of attention. Many studies investigated the natural compounds found in berries to combat diseases and promote healthy aging. This article’s scope is to indicate the potential beneficial effect of lingonberry consumption on health, to promote well-being and longevity.

## 1. Introduction

Lingonberry (*Vaccinium vitis-idaea* L.) is a small red berry of the Ericaceae family and the genus *Vaccinium*. They grow wild in Northern countries’ forests, Central Europe, Russia, and Canada [1]. The berries are mainly collected from the wild; however, some cultivars are produced on a small scale, but lingonberry plant breeding is still in its infancy [2]. The berries are consumed as food in many different ways and forms, such as raw or cooked in lingonberry jam, compote, juice, or syrup. They are a primary dietary source of anthocyanins and other phenolics for people living in the Scandinavian area [3]. Lingonberries are classed as “superfruits”, being particularly rich in antioxidants such as vitamins C, A, and E (tocopherol) and polyphenols [1,4]. The fruit is also rich in functional compounds, such as fibers and minerals [1]. Lingonberries (*Vaccinium vitis-idaea*) are closely related to cranberries (*Vaccinium oxycoccus*), but they are less known and popular than cranberries. However, in recent years, they have gained increased interest due to their high content and complex composition of phenolics and health-promoting effects. In vivo and in vitro studies have indicated various potential health beneficial effects of lingonberries, such as anti-inflammatory [5,6], antioxidant [1,6], and anticancer activities [7,8]. Traditionally they have been used for their antiseptic and antimicrobial properties [9]. Lingonberries have been shown to prevent diet-induced obesity and low-grade inflammation in diabetic animals [10,11,12]. Moreover, lingonberry intake has been associated with a beneficial health effect in preventing and treating brain aging and neurodegenerative disorders [13,14].

Despite a long cultivation history in North America and Scandinavian countries, the breeding of lingonberries is in a developmental stage. Therefore, lingonberries are not as popular and available in the marketplace as cranberries or blueberries. Lingonberry (*Vaccinium vitis-idaea* L.) is also one of the least studied fruit in the Ericaceae family [15]. Nonetheless, the health benefits and diversity of phenolics in lingonberries could provide efforts to develop and expand their commercial production successfully [16]. This review provides an overview of the current knowledge of the potential beneficial effect of lingonberry consumption on health and discusses the composition and bioavailability of lingonberry polyphenols.

## 2. Chemical Composition of Lingonberry Fruit

Lingonberry fruits are a rich source of dietary micronutrients and bioactive compounds, including vitamins, polyphenols, and minerals. Polyphenolics, such as flavonoids, polyphenolic acids, anthocyanins, procyanidins, organic acids, vitamins (A, B1, B2, B3, and C), potassium, calcium, magnesium, and phosphorous, have been found in lingonberries [3,6,17]. However, there is some variation between the content and profile of the phenolics in lingonberry fruit, depending on the region they grow in, cultivar, growing environment, ripening stage, weather, soil conditions, and extraction methods. The total phenolics content in wild lingonberry growing in Alaska was in the range of 624.4 mg/100 g FW [18], while lingonberry grown in the forests in central Poland had total phenolics in the range of 582–760 mg/100 g FW for the ethanol–water extract and 436–636 mg/100 g FW for the water extract [19]. The solubility of phenolics is higher in alcohols; thus, for the ethanol–water extract, higher results were obtained [19]. The mean concentration of the phenolic compounds in cultivated lingonberries grown in a research plot in Oregon (United States) was estimated at 566 mg/100 g (range 431–660 mg/100 g FW) [16]. Significantly lower results (360–410 mg/100 g FW) were reported for various lingonberry extracts grown in the southern Labrador area in Canada [20]. In cultivated lingonberries (US), the total anthocyanin (ACN) content ranged from 27.4 to 52.6 mg/100 g, depending on the cultivar [16], while total anthocyanins in wild fruits were in the range of 33–47 mg/100 g FW in Poland [19] and 77.5 mg/100 g FW for berries grown in Finland [21]. The highest ACN concentration accumulated in lingonberry from Alaska (194.6 mg/100 g FW) [18]. The total flavonoids content in wild lingonberry from Poland ranged from 522–647 μmol/100 g FW for the ethanol–water extract and 255–353 μmol/100 g FW for the water extract (Poland) [19]. Proanthocyanidins (PAC) exhibited the highest levels in wild Alaskan lingonberry (278.8 mg/100 g FW), which was comparable to the PAC content of the same species in Finland (260 mg/100 g FW) [18,22]. Anthocyanin glycosides, the pigments responsible for the blue and red colors in berries, are the most abundant phenolic compounds in lingonberries. Wild Alaskan lingonberry displayed only cyanidin glycosides as the dominant anthocyanin, with non-detectable levels of peonidins [18]. The higher relative content of cyanidin glycosides was linked to geographical and environmental factors in the northernmost latitudes of Finland [22]. Cyanidin-3-galactoside constitutes approximately 82.5% of all anthocyanin compounds in wild lingonberries, while cyanidin-3-arabinoside and cyanidin-3-glucoside are present in smaller amounts [6,17]. Individual anthocyanin content in cultivated lingonberries were 79% for cyanidin-3-galactoside, 10% for cyanidin-3-glucoside, and 11% for cyanidin-3-arabinoside [16]. The polyphenolic and anthocyanin contents in wild berry fruits are generally higher than in cultivated fruits. Wild berries exposed to environmental stress enhance their defenses by producing an increased number of polyphenolics, which protects plants from external agents [23]. The content of the particular classes of compounds in lingonberries depends on the location and type (wild/cultivated), as shown in Table 1. Within the group of phenolic acids, derivatives of ferulic acid, coumaric acid, caffeoylquinic acid, and benzoic acid were found in lingonberry fruit [6,17]. Moreover, flavonols, such as quercetin and its glycosylated derivatives, and two flavanols identified as catechin and epicatechin, were identified in the fruits [6]. In the aqueous extract from the fruit, the flavanol contents ranged between 30 and 36%, and the relative contents of the flavonol glycosides were in the range of 7–9%. Among the quercetin glycosides identified, quercetin-3-O-galactoside, quercetin-3-O-glucoside, quercetin rutinoside, quercetin pentosides, quercetin-3-O-rhamnoside, and quercetin-3-O-(4″-(3-hydroxy-3-methylglutaryl))-α-rhamnoside were described [1,17,24]. Additionally, kaempferol glycosides, such as kaempferol hexoside, kaempferol rutinoside, kaempferol pentoside, and kaempferol-3-O-rhamnoside, were also identified in lingonberry fruit [1,17,24]. Dimeric B-type and A-type, and trimeric proanthocyanidins type A, represent approximately 23% of the total polyphenols [6]. Wild Alaskan lingonberry exhibited a higher percentage of B-type dimers (16.5%) and trimers (12.8%) than A-type analogs (7.8 and 2.6%, respectively) [18]. Hydroxycinnamic acids represent the less abundant group of phenolic compounds in the lingonberry fruit. Their relative content was in the range of 2–3% [24]. In processed lingonberry extract, p-coumaric acid was the predominant hydroxycinnamic acid, followed by caffeic and ferulic acid. Other phenolic acids represented in the lower amount are esters of caffeic acid, chlorogenic, and crypto-chlorogenic acid. The 4-glucosides of p-coumaric and caffeic acids were also detected in a lingonberry extract [1]. Triterpenoids, lingonberry secondary metabolites, are another group of compounds with beneficial health effects. Szakiel et al. (2012) identified the main triterpenoid compounds occurring in lingonberry fruits. The quantitative determination of individual triterpenoids showed that the two isomeric acids, oleanolic and ursolic, were the most abundant compounds, comprising 70–73% of all triterpenoids in the fruit. The main lingonberry triterpenoid profile, identified by GC–MS/FID, consisted of α-amyrin, β-amyrin, betulin, campesterol, cycloartanol, erythrodiol, fern-7-en-3β-ol, friedelin, lupeol, sitosterol, stigmasterol, stigmasta-3,5-dien-7-one, swert-9(11)-en-3β-ol, taraxasterol, urs-12-en-29-al, uvaol, oleanolic acid, and ursolic acid [4].

## 3. Bioavailability of Lingonberry Polyphenols

The potential ability of foods and their bioactive compounds to exert a beneficial health effect depends on their bioavailability to target tissue or organs. In in vivo conditions, plant foods go through gastrointestinal digestion, which may cause a change or reduction in the bioactive compound content [25]. Brown et al. (2014) have demonstrated that the polyphenolic composition of lingonberry undergoes modification during digestion. Despite these changes, digested lingonberry extracts showed bioactivity [25].

The absorption and metabolism of lingonberry anthocyanins after consuming fruits were investigated in healthy subjects. Cyanidin-3-galactoside and its metabolites, cyanidin glucuronide and peonidin galactoside, a methylation product of cyanidin galactoside, were detected in the urine samples. These compounds were not detectable before the consumption of the berries. This indicates that glucuronidation and methylation are important metabolic routes when anthocyanins are consumed in whole berry products. Only trace amounts of cyanidin-3-glucoside and cyanidin-3-arabinoside were found in the urine [26]. Lehtonen et al. (2013) have found elevated hippuric acid and 4-hydroxyhippuric acid levels in urine following the ingestion of a lingonberry-enriched meal (60 g of lingonberry juice corresponding to 270 g of fresh berries). Because hippuric acid was not found in the lingonberries, it was supposed that the high benzoic acid concentration in lingonberries caused the elevation of the hippuric acid levels since it can be metabolized to hippuric acid by the liver [27]. Lingonberries are also a good source of bioavailable quercetin. In the study conducted by Erlund et al. (2003), twenty subjects consumed 100 g/day of lingonberries, black currants, and bilberries for 8 weeks. The serum quercetin concentrations were 32–51% higher compared with the control group [28]. Lehtonen et al. (2010) investigated the absorption and excretion of lingonberry flavonols in a postprandial trial by analyzing flavonol glycosides and glucuronidated flavonols in plasma, urine, and feces. Both the glycosides and glucuronides of quercetin and kaempferol glucuronides were detected in urine and plasma after the consumption of lingonberries; 14% of the flavonols in urine were glycosides and 86% were glucuronidated forms. Quercetin-3-galactoside, quercetin-3-rhamnoside, and quercetin-3-xyloside were detected in feces, quercetin-3-xyloside/-arabinoside, quercetin-3-glucuronide, and quercetin-3-rhamnoside in urine, and quercetin-3-rhamnoside, quercetin-3-glucuronide, and kaempferol-7-glucuronide in plasma [29]. Polyphenols and vitamin C are the most potential berries constituents to exert effects in vivo after consuming different berries. After intake of berries (100 g of bilberry, 50 g of lingonberry, and 100 g of black currant/strawberry puree) for 8 weeks by healthy subjects, plasma concentrations of vitamin C and the plasma concentrations of polyphenols, such as quercetin, caffeic acid, protocatechuic acid, p-coumaric acid, and vanillic acid, increased significantly in the berry group. Depending on the time point, the increases in the berry group were 51–84% for quercetin, 63–109% for caffeic acid, 21–24% for protocatechuic acid, 24–49% for p-coumaric acid, and 20–39% for vanillic acid. Vitamin C concentrations increased significantly in the berry group, from 11% to 16%, depending on the time point [30]. The bioavailability of polyphenols from berries was investigated in a randomized, placebo-controlled dietary intervention trial with 72 subjects consuming moderate amounts of berry for 8 weeks. The average intake of berries was 160 g/day (bilberries, lingonberries, black currants, and chokeberries) and the total intake of polyphenols was 837 mg/day. Plasma quercetin, p-coumaric acid, 3-hydroxyphenylacetic acid, caffeic acid, protocatechuic acid, vanillic acid, homovanillic acid, and 3-(3-hydroxyphenyl)propionic acid increased significantly in the berry group. The average increases were 51% for quercetin, 21% for protocatechuic acid, 40% for p-coumaric acid, and 31% for vanillic acid. Berry consumption also affected the plasma concentrations of other polyphenols. In the berry group, 3-(3-hydroxyphenyl)-propionic acid (33HPPA) and 3-hydroxyphenylacetic acid (3-HPAA) increased compared to the control group. The urinary excretions of quercetin, p-coumaric acid, and 3-hydroxyphenylacetic acid also increased in the berry group. This finding has shown that polyphenols from a diet containing various wild and cultivated berries are bioavailable. However, there were significant inter-individual variations in all compounds’ plasma concentrations, which could be caused by differences in the intestinal microflora of the subjects [31]. Nurmi et al. (2009) studied the metabolism of berry anthocyanins to phenolic acids in six human subjects after bilberry–lingonberry puree consumption. The berry puree contained 1435 μmol (650 mg) of anthocyanins and 339 μmol of phenolic acids. The ingested anthocyanins were detected in plasma 1.5 h after the meal, and the concentration was 138 nmol/L. The ingested amount of phenolic acids was 339 µmol while the total increase in the excretion of phenolic acids was 241 µmol. The most abundant metabolites were methylated phenolic acids (homovanillic and vanillic acids), and they were partly produced from anthocyanins [32].

## 4. Biological Activity and Health-Promoting Effects

### 4.1. Antioxidant Properties

Antioxidants’ health importance is due to their ability to protect against oxidative cell damage, leading to cancer, cardiovascular and degenerative diseases, and chronic inflammation [33]. Lingonberries, and other berries, exert beneficial health effects mainly due to their high antioxidant activity. Preliminary studies conducted by Zheng and Wang (2003) have shown that lingonberries (*Vaccinium vitis-idaea* L.) have the highest antioxidant activity among berry fruits, including blackberries, blueberries, raspberries, strawberries, and cranberries. Cyanidin 3-galactoside was the most dominant anthocyanin, contributing the most antioxidant activity in lingonberries [34]. Lingonberries had potent free radical scavenging activities for DPPH^•^, ROO^•^, ^•^OH, and O2^•-^ radicals. The lingonberry extract (50mg/mL) reduced ^•^OH and O^•-^ radicals by 83% and 99%, respectively [35].

A water extract from freeze-dried lingonberry fruit had a protective effect against oxidative stress associated with obesity-induced inflammation. The extract dose-dependently decreased intracellular reactive oxygen species (ROS) production in inflamed adipocytes by 16, 27, and 31% at concentrations of 1, 2.5, and 5 mg/mL, respectively. Quantitative PCR analysis revealed that lingonberry extracts significantly downregulated NADPH oxidase 4 (NOX4) expression, an oxidant enzyme that is one of the critical sources of intracellular ROS, and upregulated the expression of superoxide dismutase 2 (SOD2), glutathione peroxidase (GPx), and catalase in inflamed adipocytes. The extract at a dose of 5 mg/mL upregulated SOD2, catalase, and GPx genes expression by 568, 311, and 45% and inhibited NOX4 by 45% [6].

Lingonberry extract (5.8% polyphenols, 2.9% flavonols, 1.9% phenolic acids, and 1.5% anthocyanins) exhibited a significant antioxidant protective effect in animals consuming a high-fat diet (HFD) by lowering the total oxidant status by 25%. Additional intake of lingonberry at higher doses increased hepatic SOD activity. Lingonberry supplementation also significantly increased erythrocyte SOD activity in comparison to the control group. Moreover, lingonberry extract at the highest dose greatly stimulated hepatic glutathione reductase activity by 50%. Uric acid plasma concentrations were lower in groups fed HFD containing lingonberry extract, suggesting a beneficial effect on oxidative stress [1].

Only regular consumption of berries throughout the whole year can prevent or decrease cardiovascular and degenerative diseases. Lingonberries and other berries are consumed in processed forms as jam, juice (canned and frozen), syrup, and sauce after heat-processing techniques, which can decrease their antioxidant activity by one-third; thus, the excellent alternative could be dried berries [33]. The studies confirmed that both fresh and dried fruits exhibited significant antioxidant activities. During the months when fresh berry fruits are not available, dried lingonberries could be a good source of antioxidants in concentrated form. Dróżdż et al. (2017) have found that the total phenolic contents in water extracts of fresh lingonberries were 4.36 mg GE/g of raw matter, while the values obtained for dried fruit were 23.6 mg GE/g of dry weight [19]. Dried lingonberries had the total phenolics and DPPH scores much higher than their respective fresh forms due to dehydration [19,33]. When berries were dehydrated, the antioxidant capacity of lingonberries skyrocketed. Dried lingonberries had five times higher antioxidant potential than other berries products [33].

### 4.2. Anticancer Activity

There is little evidence presented for lingonberry’s anticancer effects. However, lingonberry extracts were shown to inhibit cancer cell proliferation and inhibit tumor progression in mice model systems [7,36].

In vitro studies have confirmed that lingonberry extracts inhibited the proliferation of human cervical and colon cancer cells. The viability of HeLa and CaCo-2 colon cancer cells were inhibited by lingonberries in a dose-dependent manner, with EC_50_ values of 28.7 and 38.3 μg/mL, respectively. Fractionation of the lingonberry extract showed that the anti-proliferative effect was attributed to the lingonberry tannin-rich extract, composed almost entirely of proanthocyanidins [7]. Lingonberry extract and anthocyanin fraction decreased the proliferation of both colon cancer cells HT-29 and breast cancer cells MCF-7. This inhibition correlated with the vitamin C content in the fruit and the synergistic effect of vitamin C and other substances [8]. Fermented lingonberry juice (FLJ) had an anti-invasive and anti-proliferative effect on highly proliferative and invasive oral tongue squamous carcinoma (OTSCC) cells such as HSC-3 and SCC-25. FLJ significantly inhibited the proliferation of HSC-3 cells at concentrations of 2.5 and 5.0 mg/mL [37]. JB6 P+ mouse epidermal cells pretreated with lingonberry extract produced a dose-dependent inhibition of activator protein 1 (AP-1) and nuclear factor kappa B (NF-κB) activity induced by either ultraviolet-B (UVB) or 12-O-tetradecanoylphorbol-13-acetate (TPA). TPA and UVB are carcinogens and, through ROS, stimulate AP-1 and NF-κB activities involved in the tumor progression and promotion of various types of cancers [35]. AP-1 activity induced by TPA and UVB was inhibited by 82–96% and 23–95%, and the NF-κB activity was inhibited by 55–87% or 46–97%, respectively. Lingonberry extract also blocked UVB-induced phosphorylation of the mitogen-activated protein kinase (MAPK) signaling members, such as extracellular signal-regulated protein kinase 1 and 2 (ERK1/2), p38, and MEK1/2, as well as prevented TPA-induced phosphorylation of ERK1, ERK2, and MEK1/2. Moreover, lingonberry extract induced cell apoptosis in human leukemia HL-60 cells. These data suggest that lingonberry can help prevent carcinogenesis induced by environmental carcinogens [35].

Lingonberries (glycosides of cyanidin, petunidin, peonidin, malvidin, and quercetin glycosides) also demonstrated tumor-preventive effects in animal models. Supplementation with 10% (*w*/*w*) freeze-dried lingonberries mice fed HFD for 10 weeks significantly reduced the tumor number and tumor size. Feeding with berries resulted in decreased cyclin D1 in the large adenomas and significantly inhibited the formation of intestinal adenomas, which was seen as a 15–30% inhibition in tumor number. Lingonberry prevented adenoma growth substantially by reducing tumor size by over 60% in the distal small intestine. The expression of the two genes, adenosine deaminase (ADA) and 5’ecto-nucleotidase (5-NT), were inhibited by berry treatment. Prostaglandin E_2_ (PGE2) receptor subtype EP4 expression decreased notably by lingonberry feeding, and the number and size of the colonic adenomas were significantly reduced [36].

### 4.3. Neuroprotective Activity

There is scientific evidence suggesting that a diet high in berries has positive effects on the brain and prevents age-related neurodegeneration. To exert such an effect, berries or active compounds of berries must get across the blood–brain barrier. Research has demonstrated that dietary polyphenols can be absorbed from the gastrointestinal tract and then distributed to blood and tissues and cross the blood–brain barrier [38].

In vitro studies conducted on stretch-injured cortical cell cultures prepared from neonatal rat pups have shown that the cells’ injury was completely reversed in the presence of the lingonberry fruit extract (added 15 min before injury). This suggests a high protective effect of lingonberry fruit against traumatic injury in rat brain cells [13]. Compounds identified in lingonberry extracts responsible for this effect were cyanidin-3-glucoside, cyanidin-3-galactoside, proanthocyanidin A, quercetin-3-glucoside, and quercetin-3-O-α arabinoside [13]. β-Amyloid (Aβ)-induced cell death and membrane damage in rat primary cortical and hippocampal neurons was abolished by pretreatment of cells with lingonberry polyphenol fractions (10–200 µg/mL). Flavan-3-ol- and flavonol-rich fractions reduced the intracellular Aβ levels (7–15 folds) and initiated Aβ clearance from the neurons, and significantly reduced the apoptotic caspases and acetylcholinesterase activity as compared to untreated cells [39].

Diabetes plays an essential role in developing neurologic disorders by overproducing pro-oxidant molecules and the neuroinflammatory process. In streptozotocin (STZ)-induced diabetic rats, the treatment with lingonberry (5.8% flavanols, 2.9% flavonols, 1.9% phenolic acids, and 1.5% anthocyanins) increased nucleoside triphosphate diphosphohydrolase (NTPDase) activity. Western blot analysis showed that lingonberry supplementation restored the purinergic receptors’ density to the control group’s values. Moreover, treatment with lingonberries prevented the increase in reactive species (RS) and thiobarbituric acid reactive substances (TBARS) levels in the diabetic group [14].

### 4.4. Antidiabetic, Antiobesity and Anti-Inflammatory Effects

Recent studies have shown the beneficial health effect of berry fruits in attenuating adipose tissue inflammation and insulin resistance in experimental metabolic syndrome models [6]. A study conducted on the in vitro adipose model showed the ability of the lingonberry fruit extract to suppress the inflammatory response and mitigate oxidative stress in TNF-α activated 3T3-L1 adipocytes. Lingonberry extract suppressed the expression of pro-inflammatory cytokines interleukin 6 (IL-6), monocyte chemoattractant protein 1 (MCP-1), and interleukin 1 beta (IL-1β), but significantly enhanced the anti-inflammatory cytokines expressions such as interleukin 10 (IL-10) and adiponectin. Moreover, the extract substantially inhibited leptin expression, with a significant 99% decrease at a dose of 5 mg/mL [6]. The high anti-inflammatory potential of the extract was also observed in the macrophage cell culture by downregulation of the pro-inflammatory mediators expressions, such as IL-6, tumor necrosis factor-alpha (TNF-α), IL-1β, MCP-1, cyclooxygenase-2 (COX-2), and inducible nitric oxide synthase (iNOS). The inhibitory effect of the lingonberry extract on iNOS expression and nitric oxide (NO) generation was more potent than budesonide, a glucocorticoid steroid with strong anti-inflammatory potential [6]. Hypertrophied adipocytes treated with anthocyanin and non-anthocyanin polyphenol fractions from lingonberry fruit accumulated fewer lipids and had a reduced triglyceride (TG) content. This was accompanied by the downregulated expression of the lipogenic genes involved in fatty acid and TG syntheses, such as adipocyte protein 2 (aP2), fatty acid synthase (FAS), and diacylglycerol acyltransferase-1 (DGAT1) [40]. Furthermore, in TNF-α-induced human umbilical vein endothelial cells (HUVECs), polyphenol fractions attenuated the inflammatory response by inhibiting the expression of pro-inflammatory genes (IL-6, IL-1β) and adhesion molecules such as vascular cell adhesion molecule 1 (VCAM-1), intercellular adhesion molecule 1 (ICAM-1), and selectin E (SELE) [40]. The crude extract (CE) and a polyphenol-rich fraction (PRF) from lingonberry were investigated for anti-inflammatory activity in LPS-stimulated RAW 264.7 macrophages. The polyphenol-rich fraction contained an average of 30-fold the concentration of the total phenolic compounds in the crude extracts [18]. Both CE and PRF exhibited a potent anti-inflammatory activity at a 100 ug/mL concentration with a 2-fold decrease in biomarker gene expression (IL-1β, IL-6, COX-2, iNOS) for CE and 10-fold reduction for PRF relative to the LPS-stimulated controls [18]. Lingonberry extract (benzoic acids were the most prominent identified as 1-O-benzoyl-glucose and 6-O-benzoyl-glucose) stimulated glucose uptake in human liver cells (HepG2). A significant increase in glucose uptake in the range of 42.0–48.7% was observed at the highest tested concentration (50 µg/mL). Moreover, lingonberry extract has been reported to be a potent inhibitor of α-glucosidase and α-amylase activity, with an IC_50_ ranging between 12 and 17 ug/mL [41].

Inhibition of digestive enzyme activity responsible for absorption of dietary fat and sugar from the intestine can effectively prevent obesity and type 2 diabetes. Podsędek et al. (2014) have found that among the 30 fruits examined, only lingonberry and red currant strongly inhibited pancreatic lipase activity with IC_50_ values less than 0.8 mg/mL. α-Glucosidase and α-amylase activity were also inhibited by lingonberry fruit with IC_50_ values of 102.68 mg/mL and 32.71 mg/mL, respectively [42].

The in vivo study has shown that lingonberry prevents obesity and metabolic abnormalities associated with type 2 diabetes. Mice fed HFD and supplemented with lingonberry extract (20%) for 13 weeks had lower fasting insulin levels and gained less weight than the control group. The polyphenols detected in the lingonberry extract were identified as cyanidin-3-glucoside, cyanidin-3-galactoside, cyanidin-3-arabinoside, quercetin-3-O-glucoside, quercetin-3-O-galactoside, quercetin-3-O-α-rhamnoside (quercitrin), kaempferol-deoxyhexoside, and quercetin-3-O-(4″-HMG)-α-rhamnoside. Lingonberry diet significantly decreased the body fat content, hepatic lipid accumulation, and plasma levels of the inflammatory marker plasminogen activator inhibitor-1 (PAI-1) and mediated positive glucose homeostasis effects [12]. Moreover, the total plasma cholesterol was significantly lower in the group fed lingonberry than the control group. The alanine transaminase (ALT) plasma level, a liver dysfunction marker, was significantly reduced in animals receiving lingonberries. Furthermore, animals supplemented with lingonberry had reduced liver mass and liver cholesterol content compared to the control group [12]. In a mouse model of diet-induced obesity (DIO), which closely mimics human metabolic syndrome and early type 2 diabetes linked to unhealthy lifestyle, lingonberry treatment did not affect the total body weight nor retroperitoneal and epididymal fat weight. Nevertheless, lingonberry treatment significantly decreased blood glucose levels by 28%, 25%, and 17% at 125, 250, and 500 mg/kg/d, respectively, compared to the DIO controls. Lingonberry treatment attenuated hepatic steatosis and hyperlipidemia in DIO mice. The group receiving the 250 mg/kg/d of lingonberries showed the best reduction in the steatotic histological profile [11]. Consistent with these results, lingonberry supplementation reduced liver triglyceride levels. The 125 and the 250 mg/kg/d groups demonstrated a significant reduction in liver triglycerides (39%). Moreover, the dose of 250 mg/kg/d substantially reduced total plasma cholesterol and plasma LDL by 12% and 18% compared to the control group. Lingonberry treatment at all doses increased AMP-activated protein kinase (AMPK) phosphorylation and glucose transporter type 4 (GLUT4) protein levels by 1.4- to 2-fold [11]. Lingonberry supplementation prevented the HFD-induced adverse changes in blood cholesterol and glucose levels in male C57BL/6N mice. Moreover, lingonberry prevented weight gain and epididymal fat accumulation induced by HFD. In the lingonberry-supplemented HFD group, the leptin levels were lower, and adiponectin was maintained at a normal level. An increase in the inflammatory acute phase reactant serum amyloid A (SAA) and ALT activity caused by HFD was abolished by lingonberry treatment [43]. Marungruang et al. (2018) have tested for 8 weeks on ApoE−/− mice fed HFD the health effect of whole lingonberries (wLB) and the insoluble and soluble fractions of LB supplementation. Whole lingonberries and the insoluble fraction supplementation reduced weight gain and fat deposition as well as improved the glucose response. The mice fed wLB and an insoluble fraction (insLB) showed higher plasma levels of HDL cholesterol and triglyceride levels than the HFD control. Both wLB and insLB also changed the cecal microbiota composition by decreasing the relative abundance of *Mucispillirum* and increasing *Akkermansia* [44]. Male C57BL/6 J mice fed a high-fat/high-sucrose (HFHS) diet and treated with lingonberry extract for 8 weeks had lower fasting and postprandial hyperinsulinemia, improved insulin sensitivity, and enhanced hepatic insulin clearance. Moreover, lingonberry treatment alleviated metabolic endotoxemia and intestinal inflammation linked with the change in the fecal macrobiota population (expansion of *Akkermansia Muciniphila*, *Turicibacter*, and *Oscillibacter*). In the jejunum and the colon of lingonberry-treated mice, the expression of TNF-α decreased [10]. Mice fed HFD and supplemented with 20% *w*/*w* freeze-dried whole lingonberries showed improved glycemia and liver function. Moreover, phosphatidylcholines (PC) and lysophosphatidylcholines (LPC) levels increased by 62% and 28%, and serine (Ser) and sphingomyelins (SPH) levels decreased by 13% and 26%, concomitant with reduced inflammation [45]. Kivimäki et al. (2014) investigated the cardiovascular effects of a combination of lingonberry juice and a high-salt diet (HSD) in Wistar Kyoto (WKY) rats. After a 10-week intervention, HSD induced slight inflammation in vascular endothelium and increased the heart and kidney’s size, whereas it did not increase the young WKY rats’ blood pressure. Lingonberry juice treatment partly abolished the effects of excess salt intake. The group drinking lingonberry juice had reduced biomarkers of low-grade inflammation. Lingonberry normalized elevated pro-inflammatory COX-2 expression and moderated the increase in cyclic guanosine monophosphate (cGMP), which was highly elevated in salt-loaded rats [5]. The anti-inflammatory effects of lingonberry juice were investigated in spontaneously hypertensive rats (SHR). After an 8-week intervention study, in the lingonberry groups, the aortic mRNA expressions of angiotensin-converting enzyme 1 (ACE1), COX-2, P-selectin, and MCP-1 were significantly reduced by 25% for ACE1 and 50% for COX-2, P-selectin, and MCP-1. The expression of VCAM-1 decreased by half in the lingonberry group, and plasma angiotensin II concentration was more than 50% lower than the control group [46]. Atherosclerosis-prone Apoe-/- mice fed HFD and supplemented with 44% lingonberries for 8 weeks had decreased triglyceridemia and reduced atherosclerosis. Compared to the control group, lingonberries-fed mice had a significantly lower body, liver, and epididymal fat weights. After 8 weeks, animals fed the low-fat diet and the lingonberry diet formed fewer atherosclerotic plaques in the aortic root region than the control group. Mice fed lingonberries also had lower plasma levels of total cholesterol and LDL-VLDL [47]. Besides, lingonberries caused a significant decrease in the triglyceride levels with a simultaneous increase in the HDL to LDL/VLDL ratio. The lingonberry diet also induced a significant shift in the mice’s cecal microbiota, with increased Bacteroidetes and reduced Firmicutes at the phylum level, and a decreased microbial diversity, corresponding with the lower short-chain fatty acids concentration in the cecum. Lingonberries induced increases in the species *Akkermansia muciniphila*, *Blautia producta*, *Clostridum difficile*, and *Eubacterium dolichum* [47]. Lingonberry diet prevented HFD-induced low-grade inflammation and endotoxemia and altered the gut microbiota composition. The phylum-level analysis showed that lingonberry supplementation significantly increased the relative abundance of the Bacteroides and decreased the Firmicutes. The genus *Akkermansia* increased considerably in lingonberry groups compared to the control. Moreover, the results from the jejunal gene expression analysis revealed decreased expression of LPS-sensing toll-like receptor 4 (Tlr4) and macrophage marker EGF-like module-containing mucin-like hormone receptor-like 1 (Emr1) and increased expression of the tight-junction protein-encoding occludin gene [48]. There is scientific evidence that *Akkermansia muciniphila* is associated with a beneficial health effect in obese animals by improving diet-induced insulin resistance, closely related to polyphenol-rich fruit intake. [49]. The health-promoting effects of lingonberries from in vitro and in vivo studies are summarized in Table 2.

### 4.5. Antimicrobial Properties

In plants, antimicrobial activity is mainly attributed to flavonols and flavonoids; however, secondary metabolites of plants, including tannins and terpenes, can also be responsible for their antimicrobial properties [50]. Tannins isolated from *Vaccinium vitis-idaea* L. (procyanidin B-1, procyanidin B-3, proanthocyanidin A-1, cinnamtannin B_1_, epicatechin, and catechin), showed antimicrobial activity against selected periodontal pathogens such as *Porphyromonas gingivalis* and *Prevotella intermedia*. The results showed that epicatechin-(4bU8)-epicatechin-(4bU8, 2bU*O*U7)-catechin had the strongest antimicrobial activity against *P. gingivalis* and *P. intermedia*, with an MIC of 25 µg/mL [51]. A lingonberry polyphenol-rich fraction (at 0.5–1 mg/mL) significantly reduced biofilm formation by oral streptococci *Streptococcus mutans*, *Streptococcus sobrinus*, and *Streptococcus sanguinis,* and also reduced the bioactivity of *S. mutans* (at 1–2 mg/mL) [52]. A clinical study with 30 adult participants tested fermented lingonberry juice antibacterial activity against the oral cavity pathogens *Streptococcus mutans* and *Candida.* Compared to the start point, after two weeks of lingonberry mouthwash twice daily, the *Streptococcus mutans* and *Candida* counts, visible plaque index (VPI), and bleeding on probing (BOP) were reduced [53]. Thus, lingonberries can offer a promising natural food derivative to prevent dental caries [52,53]. Ethanol, methanol, and aqueous extract from lingonberry fruit showed relatively high antimicrobial activity against *Escherichia coli*, *Micrococcus luteus*, *Pseudomonas putida*, *Bifidobacterium* spp., and *Clostridium* spp. Noteworthy, the growth of *Aspergillus niger* was stopped by ethanol and methanol lingonberry extracts. Lingonberry extracts most effectively inhibited the growth of *Proteus myxofaciens* (MIC 2 mg/mL). Aqueous extracts showed antimicrobial activity at higher concentrations (MIC > 4 mg/mL) [50]. Lingonberries also possess antifungal properties and have been used in treating fungal infections. *Candida glabrata* is an opportunistic fungal pathogen that causes serious infections, particularly in people with a suppressed immune system. Pärnänen et al. (2017) studied the effect of fermented lingonberry juice (FLJ) on *C. glabrata* intracellular protein expression. They found that FLJ affects the intracellular stress response in *C. glabrata*, impairing its ability to express proteins related to oxidative stress or maintaining cell wall integrity. FLJ, through reduced energy supplies involved in the pathological and excessive biofilm accumulation, decreased the pro-pathogenic potential of *C. glabrata* [54].

Because of consumer demands, the food industry searches for safe and environmentally friendly food preservatives. Lingonberry concentrate added to commercial sugar-reduced fruit spreads was tested for antifungal activities against *Absidia glauca*, *Penicillium brevicompactum*, *Saccharomyces cerevisiae*, and *Zygosaccharomyces bailii*, as well as xerophilic environmental isolates of *Penicillium* and *Eurotium*. Because of the high concentration of benzoic acid (1.78 g/kg) and p-hydroxybenzoic acid (30.24 mg/kg), the lingonberry concentrate inhibited visible colonies’ growth of several fungi in sugar-reduced fruit. *A. glauca* and P. *brevicompactum* were the most sensitive fungi to sodium benzoate and the lingonberry concentrate [55].

Importantly the methanol extract from freeze-dried lingonberry has shown antiviral activity. The extract markedly inhibited the replication of coxsackievirus B1 (CV-B1) and influenza A virus, and anthocyanin fractions strongly inhibited the replication of influenza virus A/H3N2 [56]. There are limited licensed and applied therapeutic agents for the therapy of influenza and other human enteroviral infections. Therefore, the search for novel natural antiviral agents against viral infections is essential. Berries such as lingonberry could be a valuable resource of promising antiviral compounds, and they deserve further in-depth investigation [56]. Table 3 summarizes lingonberries’ antimicrobial activity.

### 4.6. Antioxidant Capacity and Bioactive Compounds Content in Lingonberry Food Products

Because the season during which fresh lingonberries are available is short, only a small proportion of berries is consumed fresh. Most of them are preserved by freezing or by processing them into juices, jams, and jellies. Häkkinen et al. (2000) studied the effects of domestic processing and storage on the flavonols and vitamin C content in commonly consumed berries in Finland. The fresh lingonberries were crushed and stored in their juice in a refrigerator. After 24 h at +5 °C, the level of quercetin decreased by 40% in lingonberries, probably due to enzymatic reactions that start when the cellular compartment breaks down. Despite the loss, the freshly crushed berries had a high level of quercetin (100 mg/kg). During 6 months of storage in a refrigerator, the level of quercetin remained relatively stable in crushed lingonberries, probably due to the slowing down of the enzymatic and oxidative reactions [57]. In juices prepared using standard domestic processing methods, reductions in the flavonol content were observed. In lingonberry juice (unpasteurized), only 15% of quercetin was extracted into the juice. While making juice, the skins are removed by filtering, and flavonols are concentrated mainly in the fruit skins. The quercetin level was higher (up to 45%) when juice was prepared by cold-pressing. Cooking also resulted in quercetin loss, probably due to the breakdown of quercetin during the cooking and oxidative reactions [36]. Various processing methods applied to food materials may affect the polyphenol content and also have significant effects on their antioxidant capacity. Dinstel et al. (2013) conducted research to test the antioxidant levels on home-processed lingonberry products. Using recommended recipes from the Alaska Cooperative Extension, frozen lingonberries were processed into jam, juice (canned and frozen), syrup, sauce, fruit leather, and dried fruit. The ORAC value for fresh lingonberry (203 µMTE/g) was the highest compared to others tested berries. The ORAC value of the frozen fruit was slightly lower and reached 160 µMTE/g. In canned berry products, the ORAC value decreased by half to 120 µMTE/g for canned juice and 99 µMTE/g for canned fruit. After heat-processing techniques, a considerable loss in antioxidant potential was observed. The ORAC values for sauce, syrup, and jam decreased to 51, 44, and 36 µMTE/g, respectively. When lingonberries were dehydrated, the ORAC values skyrocketed. Dried lingonberries had an ORAC score of 850 µMTE/g, while skin leather reached 550 µMTE/g [33]. Most home processing methods reduced the polyphenol and antioxidant levels; thus, when fresh berries are not available, dried fruit consumption should be recommended as an essential source of high antioxidant levels in each gram of product [33].

## 5. Conclusions

Berries, especially members of the Ericaceae family, are among the best dietary sources of bioactive compounds with beneficial health effects. The popular berries are in the genus *Vaccinium*, such as bilberry (*Vaccinium myrtillus*), cranberry (*Vaccinium macrocarpon, V. oxycoccos*), and blueberry (*Vaccinium corymbosum*). Lingonberry (*Vaccinium vitis-idaea*) is less prevalent in the daily human diet because they are collected from the wild, and plant breeding of lingonberry is still on a small scale. However, lingonberry has the highest antioxidants content among berries and possesses a broad range of health-promoting effects. The introduction of lingonberries to the daily diet can have a positive health effect, especially for patients suffering from civilization diseases, which are widespread among people worldwide. To prevent or alleviate the civilization diseases or chronic “lifestyle” diseases, such as obesity, type 2 diabetes, atherosclerosis, and hypertension, a daily diet change is necessary. This change should be “chronic” as well. Only regular consumption of berries throughout the whole year can prevent or decrease cardiovascular and degenerative diseases. Fresh lingonberry fruit is the best source of bioactive compounds and antioxidants, but they are not available on the market all year round. Therefore, the consumption of dried berries, which by removing the moisture makes the skin and pulp concentrated and increases the antioxidant levels in each gram of product, in the form of teas or snacks, could be part of a healthy lifestyle.

## Figures and Tables

**Table 1:** Mean total phenolics and monomeric anthocyanins content in wild and cultivated lingonberries.

Type and Locality	Total Phenolics (mg/100g FW)	Total Anthocyanins (mg/100g FW)	Ref.
Poland (wild)	598	40.5	[3,19]
Alaska US (wild)	624	194	[18]
Oregon US (cultivated)	566	40	[16]

**Table 2:** Biological activity and the health effect of lingonberry fruit.

Source and Treatment	Type of Model	Effects	Reference
Antioxidant Activity			
Freeze-dried lingonberry extract (1–5 mg/mL)	3T3-L1 adipocytes	↓ROS production, ↓NOX4, ↑SOD2, ↑GPx, ↑catalase	[6]
Lingonberry extract (23 mg/kg of body weight) for 42 days	Rats fed HFD	↓Total oxidant status, ↑hepatic and erythrocyte SOD, ↑hepatic glutathione reductase, ↓uric acid plasma concentration	[1]
**Anticancer Activity**			
Lingonberry extract (proanthocyanidins)	HeLa and Caco-2 cells	↓Cancer cell proliferation	[7]
Lingonberry extract (quercetin, quercetin glycosides, benzoic acid, ellagic acid) and anthocyanin fraction	HT-29 and MCF-7 cells	↓Cancer cell proliferation	[8]
Fermented lingonberry juice (2.5–5.0 mg/mL)	HSC-3 and SCC-25 cells	Anti-proliferative and anti- invasive effect	[37]
Lingonberry extract (0.28 mg/g anthocyanins, 0.95 mg/g phenolics)	JB6 P+ mouse epidermal cells	↓AP-1 and NF-*k*B activity, ↓MAPK phosphorylation, ↓ERK1, ↓ERK2, ↓p38, and ↓MEK1/2 kinase	[35]
Lingonberry extract	HL-60 cells	Induced cell apoptosis	[35]
Freeze-dried lingonberry (10% *w*/*w*; 472 mg/kg total anthocyanins, 97 mg/kg total flavonols)	Mice fed HFD	↓Intestinal adenomas formation, ↓ tumor number and size, ↓ADA and 5-NT expression, ↓cyclin D1, ↓PGE2	[36]
**Neuroprotective Activity**			
Lingonberry extract (1 µL)	Cortical cell cultures from neonatal rat pups	Protected from cells injury	[13]
Lingonberry polyphenol fraction	Primary cortical and hippocampal neurons	↓β-Amyloid levels, ↓ AChE activity, ↓ Apoptotic caspases	[39]
Lingonberry extract for 30 days	Diabetic rats	↑NTPDase activity, restored density of purinergic receptors, ↓RS, ↓ TBARS	[14]
**Antidiabetic, antiobesity and anti-inflammatory activity**			
Freeze-dried lingonberry extract (1–5 mg/mL)	3T3-L1 adipocytes	↓IL-6, ↓MCP-1, ↓IL-1β, ↓leptin, ↑IL-10, ↑adiponectin	[6]
Lingonberry anthocyanin and non-anthocyanin polyphenol fractions (5–20 µg/mL)	3T3-L1 adipocytes	↓Lipid accumulation, ↓TG content, ↓aP2, ↓FAS, ↓DGAT1	[40]
Lingonberry non-anthocyanin polyphenol fraction (0.1–10 µg/mL)	HUVECs	↓IL-6, ↓IL-1), ↓VCAM-1, ↓ICAM-1, ↓SELE	[40]
Freeze-dried lingonberry extract (0.05–1 mg/mL)	RAW 264.7 macrophages	↓IL-6, ↓TNF-α, ↓IL-1β, ↓MCP-1, ↓COX-2, ↓iNOS, ↓NO generation	[6]
Lingonberry crude extract and polyphenol-rich fraction	RAW 264.7 macrophages	↓IL-6, ↓IL-1β, ↓COX-2, ↓iNOS,	[18]
Lingonberry extract (12.5–50 µg/mL, benzoic acids)	HepG2 cells	↑Glucose uptake, ↓ α-glucosidase and ↓α-amylase activity	[41]
Freeze-dried lingonberry extract (20% *w*/*w*) for 13 weeks	C57BL/6J mice fed HFD	↓Body fat and hepatic lipid, ↓fasting insulin, ↓PAI-1 and ↓ALT plasma levels, ↓total cholesterol	[12]
Lingonberry (freeze-dried) ethanol extract (125, 250, 500 mg/kg) for 8 weeks	Mice with diet-induced obesity (DIO)	↓Blood glucose levels, ↓hepatic steatosis, ↓hyperlipidemia, ↓liver triglyceride, ↓total plasma cholesterol, ↓LDL level, ↑GLUT4 expression, ↑AMPK phosphorylation	[11]
Air-dried lingonberry powder (20% *w*/*w*) for 6 weeks	C57BL/6J mice fed HFD	↓Weight gain, ↓epididymal fat, ↓blood cholesterol, ↓glucose level, ↓leptin, ↑adiponectin, ↓inflammatory markers (SAA)	[43]
Lingonberry fruit and insoluble fraction of lingonberries for 8 weeks	ApoE−/− mice fed HFD	↓Weight gain and fat deposition, ↑HDL cholesterol, changed the cecal microbiota composition (↓*Mucispillirum*, ↑A*kkermansia)*	[44]
Lingonberry extract (200 mg extract/kg body weight) for 8 weeks	C57BL/6J mice fed HFHS	↓Fasting and postprandial hyperinsulinaemia, ↑insulin sensitivity, ↓metabolic endotoxaemia, ↓intestinal inflammation (↑*Akkermansia*, ↑*Turicibacter*, ↑ *Oscillibacter*)	[10]
Freeze-dried lingonberry (20% *w*/*w*)	Mice fed HFD	↑Glycemia and liver function, ↑PC, ↑LPC, ↓serine, ↓SPH	[45]
Lingonberry juice for 10 weeks	Rats fed high salt diet	↓Biomarkers of low-grade inflammation, ↓COX-2 expression	[5]
Lingonberry juice for 8 weeks	SHR rats	↓ACE1, ↓COX-2, ↓P-selectin, ↓MCP-1, ↓VCAM-1, ↓angiotensin II	[46]
Dried lingonberry fruit (44% in diet) for 8 weeks	Apoe-/- mice fed HFD	↓Body weight gain, ↓liver and epididymal fat weights, ↓atherosclerotic plaques, ↓total cholesterol, ↓LDL-VLDL, ↑HDLcholesterol, change in cecal microbiota (↑*Bacteroidetes*, ↓*Firmicutes*)	[47]
Freeze-dried lingonberry (20% *w*/*w*) for 11 weeks	C57BL/6J mice fed HFD	↓Low-grade inflammation, ↓endotoxemia, change in gut microbiota (↑*Bacteroidetes, ↓Firmicutes*)	[48]

↑ Increase; ↓ Decrease. Abbreviations used in Table 2 ACE1, angiotensin-converting enzyme 1; AChE, acetylcholinesterase; AMPK, AMP-activated protein kinase; AP-1 activator protein 1; ADA, adenosine deaminase; aP2, adipocyte protein 2; ALT, alanine transaminase; SAA, acute phase reactant serum amyloid A; COX-2, cyclooxygenase-2; DGAT1, diacylglycerol acyltransferase-1; 5-NT, 5’ecto-nucleotidase; ERK1/2, extracellular signal-regulated kinase 1/2; FAS, fatty acid synthase; GLUT4, glucose transporter type 4; GPx, glutathione peroxidase; HDL, high-density lipoprotein; iNOS, inducible nitric oxide synthase; ICAM-1, intercellular adhesion molecule 1; IL-1 β, interleukin 1 beta; IL-6, interleukin 6; IL-10, interleukin 10; LDL, low-density lipoprotein; LPC, lyso-phosphatidylcholine; MAPK, mitogen-activated protein kinase; MEK1/2, mitogen-activated protein kinase kinase 1/2; MCP-1, monocyte chemoattractant protein 1; NOX4, NADPH oxidase 4; NO, nitric oxide; NF-κB, nuclear factor kappa B; NTPDase, nucleoside triphosphate diphosphohydrolase; p38, p38 mitogen-activated protein kinases; PC, phosphatidylcholine; PAI-1, plasminogen activator inhibitor-1; PEG2, prostaglandin E_2_; ROS, reactive oxygen species; RS, reactive species; SELE, selectin E; SPH, sphingomyelins; SOD, superoxide dismutase; TBARS, thiobarbituric acid reactive substances; TG, triglyceride; TNF-α, tumour necrosis factor alpha; VCAM-1, vascular cell adhesion molecule 1; VLDL, very-low-density lipoprotein.

**Table 3:** Antimicrobial activities of lingonberry fruit polyphenols.

Source	Microorganism	Effects	Reference
Tannins from lingonberries	*Porphyromonas gingivalis Prevotella intermedia*	Inhibited bacteria growth with MIC 25 µg/mL	[51]
Lingonberry polyphenol rich fraction (586.8 mg/g total polyphenols and 374.6 mg/g total flavanols)	*Streptococcus mutans*, *Streptococcus sobrinus*, *Streptococcus sanguinis*	Reduced biofilm formation	[52]
Fermented lingonberry juice (mouthwasch)	*Streptococcus mutans Candida*	Reduced visible plaque index and bleeding	[53]
Lingonberry extracts (methanol, ethanol, water)	*E. coli*, *M. luteus*, *P. putida*, *P. myxofaciens*, *Clostridium* sp., *Bifidobacterium* *sp., A. niger*	Inhibited bacteria growth (MIC 2–4 mg/mL)	[50]
Fermented lingonberry juice	*Candida glabrata*	Reduced biofilm formation	[54]
Comercial lingonberry concentrate (benzoic acid, p-hydroxybenzoic acid)	*Absidia glauca, Penicillium brevicompactum, Saccharomyces cerevisiae Zygosaccharomyces bailii, Penicillium* and *Eurotium*	Inhibited the growth of fungi (3–24% lingonberry conc.)	[55]
Methanol extract from freeze-dried lingonberries (flavonoid and phenolic fractions)	Coxsackievirus B1 (CV-B1) and influenza virus A/H3N2	Inhibited viruses replication (IC_50_ 100–800 µg/mL)	[56]
